# Behavioral and Transcriptional Response to Selection for Olfactory Behavior in *Drosophila*

**DOI:** 10.1534/g3.120.401117

**Published:** 2020-02-05

**Authors:** Elizabeth B. Brown, John E. Layne, Alexandra R. Elchert, Stephanie M. Rollmann

**Affiliations:** Department of Biological Sciences, University of Cincinnati, OH 45221

**Keywords:** olfactory behavior, valence, chemosensation, *Drosophila melanogaster*, RNA-seq, Glutathione S-transferase

## Abstract

The detection, discrimination, and behavioral responses to chemical cues in the environment can have marked effects on organismal survival and reproduction, eliciting attractive or aversive behavior. To gain insight into mechanisms mediating this hedonic valence, we applied thirty generations of divergent artificial selection for *Drosophila melanogaster* olfactory behavior. We independently selected for positive and negative behavioral responses to two ecologically relevant chemical compounds: 2,3-butanedione and cyclohexanone. We also tested the correlated responses to selection by testing behavioral responses to other odorants and life history traits. Measurements of behavioral responses of the selected lines and unselected controls to additional odorants showed that the mechanisms underlying responses to these odorants are, in some cases, differentially affected by selection regime and generalization of the response to other odorants was only detected in the 2,3-butanedione selection lines. Food consumption and lifespan varied with selection regime and, at times, sex. An analysis of gene expression of both selection regimes identified multiple differentially expressed genes. New genes and genes previously identified in mediating olfactory behavior were identified. In particular, we found functional enrichment of several gene ontology terms, including cell-cell adhesion and sulfur compound metabolic process, the latter including genes belonging to the glutathione S-transferase family. These findings highlight a potential role for glutathione S-transferases in the evolution of hedonic valence to ecologically relevant volatile compounds and set the stage for a detailed investigation into mechanisms by which these genes mediate attraction and aversion.

Animals encounter volatile chemical cues in the environment. These chemicals can prompt attractive behavior, such as approaching a food source, a potential mate, or an oviposition site. They can also provoke avoidance behavior, such as avoiding toxins or predators (Depetris-Chauvin *et al.* 2015; Mansourian and Stensmyr 2015; Hansson and Wicher 2016). The successful detection and subsequent response to such environmental cues is essential to reproduction and survival. To appreciate this vital connection, it is fundamentally important to understand the translation of odorant detection into behavioral output, including the genetic/neural factors that underpin attractive/aversive olfactory behavior (Joseph and Carlson 2015). Although substantial advances have been made in identifying the neural circuity underling the detection of chemical cues (Masse *et al.* 2009; Wilson 2013; Groschner and Miesenböck 2019), the genetic contributions to aversive and attractive olfactory behavior remains a long-term goal in the field of behavioral genetics.

The vinegar fly *Drosophila melanogaster* has emerged a model system for elucidating the genetic basis of olfactory behavior; significant progress has been made in revealing the neural circuitry that underlies the peripheral detection of chemical cues. In combination with our understanding of their chemical ecology, *Drosophila* represent an excellent system in which to investigate the evolution of hedonic valence to ecologically relevant volatile compounds. Among the many odorants present in the environment, flies are particularly attracted to volatiles emitted as a by-product of yeast fermentation. Indeed, it is these volatiles, and not those emitted by the fruit itself, that mediate attraction to and oviposition on a food substrate (Becher *et al.* 2012). Fermenting fruit may also contain potentially harmful bacteria and/or microbes (Castillo *et al.* 1999; Stensmyr *et al.* 2012), and volatile compounds emitted by these can elicit robust avoidance behavior.

Odorants bind to receptor proteins embedded in the dendrites of olfactory sensory neurons (OSNs), which are located inside sensilla on either the third antennal segment or the maxillary palp. These dendrites are bathed in an aqueous lymph in which is suspended both odorant degrading enzymes and odorant binding proteins. These latter proteins have been shown to influence insect perception and are hypothesized to eliminate or transport odorants in the sensillar lymph (Galindo and Smith 2001; Hekmat-Scafe *et al.* 2002; Larter *et al.* 2016). There are two families of receptor proteins: odorant receptors (ORs) and ionotropic receptors (IRs). The OR gene family together with the highly conserved co-receptor, ORCO, are hypothesized to form ligand-gated ion channels for the detection of environmental volatiles (Clyne *et al.* 1999; Vosshall *et al.* 2000; Robertson *et al.* 2003; Larsson *et al.* 2004; Sato *et al.* 2008, Wicher *et al.* 2008). The IRs, derived from ionotropic glutamate receptors, have also been implicated in odor detection, primarily of acids and amines (Benton *et al.* 2009; Abuin *et al.* 2011). The OSNs project axons to distinct glomeruli in the antennal lobe (Laissue *et al.* 1999; Vosshall *et al.* 2000; Dobritsa *et al.* 2003; Hallem and Carlson 2004; Couto *et al.* 2005). Within each glomerulus, the olfactory neurons synapse onto two second order neurons: local interneurons which inhibit olfactory neuron axons terminating in other glomeruli, and second order projection neurons (PNs) which carry sensory information that has been modified by this inhibitory process via axons to the mushroom body and lateral horn regions of the brain (Marin *et al.* 2002; Wong *et al.* 2002; Tanaka *et al.* 2004; Wilson *et al.* 2004; Jefferis *et al.* 2007). This entire pathway can be thought of as a series of sensory channels, carrying the spatial and temporal pattern of antennal lobe activity, which allows discrimination among the diverse odorants present in the environment (Gao *et al.* 2000; Vosshall *et al.* 2000; Wang *et al.* 2002).

Odor processing channels that evoke highly stereotyped aversion and attraction responses have been identified for specific odors of ecological significance. For example, low concentrations of apple cider vinegar elicit attraction as well as responses from OR42b- and OR92a-expressing neurons, while high vinegar concentrations elicit aversion upon recruiting additional OR85a-expressing neurons (Semmelhack and Wang 2009). Dedicated ORs have also been found that mediate attraction to amines (IR92a; Min *et al.* 2013) and farnesol (OR83c; Ronderos *et al.* 2014), as well as aversion to acids (IR64a; Ai *et al.* 2010) and to the microbe-associated odor geosmin (OR56a; Stensmyr *et al.* 2012). Such studies suggest, at least for some odors, that hedonic valence is correlated with the activity of single odor processing channels. However, most ORs do not display such a high degree of ligand specificity (Malnic *et al.* 1999). In fact, odor perception is thought to be most commonly determined by the combined activity of multiple OSN classes. Recent literature suggests that that this is indeed the case; behavioral responses result from patterns of glomerular responses and not necessarily single specialized glomeruli (Badel *et al.* 2016). Furthermore, representations of odor valence have been shown to spatially segregate, with attractive and aversive odor cues activating predominantly medial-projecting and lateral-projecting PNs, respectively (Knaden *et al.* 2012; Knaden and Hansson 2014).

To gain insight into how the olfactory system mediates hedonic responses to ecologically relevant odors, we conducted artificial selection experiments for thirty generations in which we independently selected for positive and negative behavioral responses to two chemical compounds: 2,3-butanedione and cyclohexanone. These compounds are a natural byproduct of yeast fermentation (de Carvalho *et al.* 1991; Mayr *et al.* 2003; Birch *et al.* 2013; Krogerus and Gibson 2013), a mediator of *Drosophila* attraction and oviposition site preference (Becher *et al.* 2012). We also examined the extent to which the response to selection was odorant specific by measuring the olfactory behavior of lines from both selection regimes to a battery of additional odorants. We then performed RNA-seq to identify changes (if any) in gene expression among the artificially selected lines.

## Materials and Methods

### Drosophila maintenance and husbandry

All flies were maintained in bottles on standard cornmeal/agar/molasses media at 25° under a 12:12 hr light-dark cycle. The DGRP-derived advanced intercross population was provided by Dr. Trudy F.C. Mackay (Huang *et al.* 2012). This population was created by crossing 40 lines of the *Drosophila* Genetic Reference Panel (DGRP) in a round-robin design. A single male and female from each cross were placed into each of 10 bottles. For each generation afterward, a subset of flies from each bottle were placed into each of 10 bottles to seed the subsequent generation, such that the census size of this population is N = 800.

### T-maze assay

All behavioral assays were conducted as previously described (Brown *et al.* 2017). Briefly, 30 flies were placed into the center of a T-maze apparatus. After a 1 min acclimation period, flies were given 1 min to choose between one arm of the maze containing the diluted odorant or one arm containing the vehicle only. The number of flies in each arm were counted and then a preference index (PI) was calculated using the formula: PI = (O - N) / (O + N), where O is the number of flies on the odor side and N is the number of flies on the side not containing odor. A positive PI indicates attraction to the odor, whereas a negative PI indicates repulsion. All assays were conducted in the morning, in the dark, at 25° on virgin male and female flies aged 3-7 days post-eclosion. Prior to testing, flies were food-deprived for 14-18 hr on 1% agar (MoorAgar Inc., Rocklin, CA). Each line and sex were tested separately.

### Artificial selection and measurements of olfactory behavior

We artificially selected for positive and negative behavioral responses to 2,3-butanedione (BUT) and cyclohexanone (CYC; Sigma-Aldrich; St. Louis, MO). All behavioral assays were performed using 2,3-butanedione at 0.1% and using cyclohexanone at 0.01%, unless otherwise noted. Artificial selection experiments were performed as previously described (Brown *et al.* 2017). Briefly, behavioral responses to either BUT or CYC were measured in the T-maze as described in the previous section. After each assay, flies from the odor side and non-odor side were collected and maintained separately. Flies collected from the odor side of the T-maze were used to establish the PI^+^ lines, while files from the non-odor side were used to establish the negative PI^-^ lines. Odor-guided behavior was tested until a minimum of 25 females and 25 males were obtained for each of three replicate positive PI (PI^+^) and three negative PI (PI^-^) lines for each selected odorant (6 lines × 2 odorants). Herein we refer to these lines by the odorant used in a given selection regime (BUT or CYC) and the directionality of selection (positive/negative PI; *e.g.*, BUT^+^ or BUT^-^ lines, as specific cases of the generic PI^+^ and PI^-^ terms). To establish the control lines, behavioral assays were conducted in which there was no odor present on either side of the T-maze and flies were collected from one randomly selected side. Three replicate control lines were also generated. This selection regime was repeated for 30 generations. Estimates of realized heritability (*h*^2^) were calculated by regression of the cumulative response to selection against the cumulative selection differential (Falconer and Mackay 1996).

### Assessment of other correlated organismal traits

Additional measurements of olfactory behavior, including a test for symmetrical responses to selection, an assessment of behavioral responses to other odorants, and dose-dependent shifts in olfactory behavior were conducted using the same methods as described above. Locomotor reactivity was measured as described previously (Jordan *et al.* 2006). Briefly, locomotor reactivity was measured as the amount of time a single fly is active within 45 sec immediately following a mechanical disturbance. For each line and sex, 10 replicate measurements were taken on mated flies aged 3-5 days post-eclosion. For longevity and starvation resistance measurements, first instar larvae were transferred into vials at a constant density of 50 individuals per vial (Leips and Mackay 2002). To measure longevity, adults were separated by sex and placed into vials containing 10 flies each and then scored every 24 hr (Linford *et al.* 2013). Every 2-3 days, flies were transferred to fresh media and dead flies removed. To measure starvation resistance, adults were separated by sex and then placed on 1% agar (Harbison *et al.* 2004). Survival was then measured every 8 hr. For each experiment, eight replicate vials were measured for each line and sex. Food consumption was measured using the CAFE assay (Ja *et al.* 2007; Deshpande *et al.* 2014). Briefly, five flies were placed into a vial containing 1% agar as a water source and 5 μL of liquid food within a calibrated glass micropipette (VWR, Radnor, PA). The liquid food was composed of 5% sucrose (Sigma-Aldrich) and 5% yeast extract (Fisher Scientific, Hampton, NH). Flies were habituated for 24 hr prior to testing and then food consumption was quantified during the following 24 hr. To assess evaporation, vials without flies were maintained, and the amount of food consumed was adjusted accordingly. Measurements were taken on mated flies aged 3-5 days post-eclosion. For each line and sex, 12 replicate vials were tested.

### Assessment of physicochemical similarity

For each selection regime, behavioral responses to novel odorants were measured in each of the three PI^+^ and PI^-^ lines, as well as the three control lines. A total of 12 odorants were tested at a concentration of 0.01% and include: ethyl lactate, pentyl acetate, 2-pentanone, 2-heptanone, hexyl acetate, ethyl butyrate, 2-butanone, 6-methyl-5-hepten-2-one, ethyl hexanoate, propyl acetate, acetone, and acetophenone. To assess generalization of valence from selected to similar but novel molecules, we computed the “physicochemical distance” between the selected and novel, a multidimensional metric that integrates over 1600 physical properties (Haddad *et al.* 2008). For each selection regime, we regressed the ΔPI* against the physicochemical distance between the selection and test odorants, where ΔPI^+^ = $\bar{x}$_PI+_ - $\bar{x}$_ctrl_, and ΔPI^-^ = $\bar{x}$_ctrl_ - $\bar{x}$_PI_- ($\bar{x}$ is the mean of the replicates).

### Statistical analysis

For measurements of olfactory behavior, locomotion, food consumption, lifespan, and starvation resistance, we performed a nested mixed model analysis of variance (ANOVA), where Y = μ + Selection + Line (Selection) + Sex +Selection x Sex + Line (Selection) x Sex + ε. Selection is the fixed effect of selection treatment (PI^+^, PI^-^, or control), Line is the random effect of replicate within each selection regime, Sex is the fixed effect of sex, and ε indicates error. If no significant difference between sexes was observed, the data were pooled. *Post-hoc* analyses were conducted using Tukey’s HSD test. For the analysis of physicochemical similarity, we performed a linear regression. All data were analyzed using JMP 12.0 software (SAS Institute Inc., Cary, NC).

### RNA isolation and sequencing

Whole heads from 100 female adult flies, aged 3-7 days post-eclosion, were dissected on dry ice in the morning. Two independent biological samples were collected for each of the three replicate PI^+^ and PI^-^ selection lines for each odor as well as from the three unselected control populations. Collection of each sample was randomized and occurred over a five-day period. Heads were mechanically crushed using RNase-free pestles and total RNA was isolated using the RNeasy Mini Kit (Qiagen, Valencia, CA). Total RNA was sent to the Weill Cornell Medical College Genomics Resources Core Facility for RNA sequencing using standard protocols. There, cDNA libraries were generated from each sample and then sequenced using Illumina HiSeq4000 to generate 100 bp reads.

### RNA-Seq processing and analysis

Raw sequencing data were filtered for adapter contamination using the program Trim Galore! (http://www.bioinformatics.babraham.ac.uk/projects/trim_galore), allowing for a maximum error rate of zero. The Cutadapt program was then used to trim low quality sequences that have Phred scores below 20 as well as remove reads shorter than 30 bp from the analysis (Martin 2011). The remaining RNA-seq reads were then aligned to the *Drosophila melanogaster* reference genome (version 6.10; Attrill *et al.* 2016) using STAR (Dobin *et al.* 2013). Differentially expressed genes between selection regimes were identified using the Bioconductor EdgeR package (Robinson *et al.* 2010). Raw read counts were filtered so that only genes that contain at least 1 read per million in at least half the samples were used for subsequent analyses. The data were then normalized for library size using the *calcNormFactors function*. To identify differential gene expression among the PI^+^, PI^-^, and the control treatments, three comparisons were performed for each odorant: (1) PI^+^ *vs.* control, (2) PI^+^ *vs.* PI^-^, and (3) PI^-^ *vs.* control. Rather than statistically nesting lines within the selection groups, the lines from each group were pooled for each pairwise comparison in order maximize detection of differences in gene expression. To account for multiple testing, we applied a FDR of 0.1.

### Functional annotation

The program Panther was used determine overrepresentation of Gene Ontology (GO) terms (Mi *et al.* 2013; Mi *et al.* 2017; Mi *et al.* 2019). A total of six lists were composed, of genes that survived the FDR of 0.1 from each pairwise comparison, as mentioned above. Each gene list was then separately provided to the software and annotated. For functional annotation of each dataset was done with the PANTHER GO Slim function (reference organism: *Drosophila melanogaster*). The statistical parameters were Fisher’s Exact test combined with a False Discovery Rate correction. The PANTHER-suggested defaults were used for all other options, including the nature of annotations used.

### Data availability

The RNA-seq data reported in this paper have been deposited on the National Center for Biotechnology Information (NCBI) Gene Expression Omnibus under accession number GSE144433 and is publicly accessible at https://www.ncbi.nlm.nih.gov/geo/. All other data necessary to confirm the conclusions presented in this article are present within this article, figures, tables, and supplemental materials. Supplemental material available at figshare: https://doi.org/10.25387/g3.9956633.

## Results

### Artificial selection for olfactory behavior

**Figure 1:**
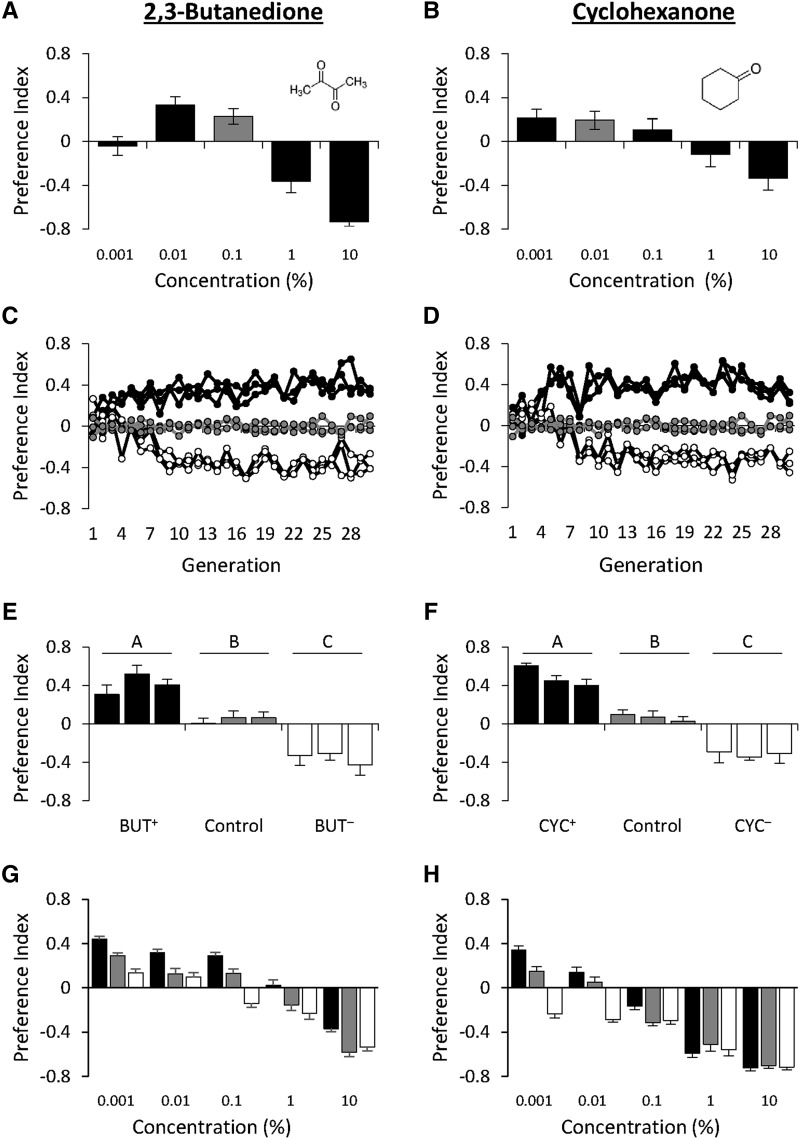
Behavioral responses to 2,3-butanedione or cyclohexanone. (A and B) Dose response curves of the base population to (A) 2,3-butanedione and (B) cyclohexanone. Gray bars indicate the concentration used for selection. Data shown are means ± SE. N = 20. (C and D) Response to selection for attractive and aversive behavioral responses to (C) 2,3-butanedione and (D) cyclohexanone. Mean PI at each generation for the three replicate PI^+^ lines (black dots), three PI^-^ lines (white dots), and three control lines (gray dots) are shown. N = 6. (E and F) Assessment of divergent selection for behavioral responses to (E) 2,3-butanedione and (F) cyclohexanone relative to control. Data shown are means ± SE. Letters indicate *P* < 0.05 using Tukey’s *post hoc* test. N = 20. (G and H) Dose response curves of each selection regime after selection for behavioral responses to (G) 2,3-butanedione and (H) cyclohexanone. At each concentration, mean PI of one representative PI^+^ (black bars), PI^-^(white bars), and control treatment (gray bars) are shown. Data shown are means ± SE. N = 20.

To understand the mechanisms underlying olfactory behavior, we performed artificial selection experiments using the DGRP-derived advanced intercross population, an outbred base population derived from the *Drosophila* Genetic Reference Panel (DGRP; Huang *et al.* 2012). Dose response curves to two odorants, 2,3-butanedione (BUT) and cyclohexanone (CYC), yielded concentration-specific differences in behavioral responses (Figure 1A, B; Supplemental material, Table S1) and based on these, we selected a single odorant concentration with which to carry out artificial selection: 0.1% for BUT and 0.01% for CYC. For each odorant, we applied artificial selection and generated three replicate lines for positive PI responses and three for negative (Figure 1C, D; Supplemental material, Table S2). Three replicate unselected control lines were also generated. Behavioral responses to each odorant were measured for the two selection groups (^+^/^-^) and the control. Bisymmetrical responses to selection were found under both selection regimes, with significant differences between the selection treatments and the control (Figure 1E, F; Supplemental material, Table S3). The BUT^+^ treatment had an average PI of 0.41 (± 0.08 SE), the BUT^-^ treatment -0.36 (± 0.09), and the control treatment 0.05 (± 0.06). The CYC^+^ treatment averaged 0.065 (± 0.06), the CYC^-^ -0.31 (± 0.08), and the control 0.07 (± 0.06). These differences are not the result of a general change in locomotion because there was no significant difference in locomotor reactivity among the selection treatments (Supplemental material, Figure S1; Supplemental material, Table S4).

For each selected odor, we next estimated the realized heritability (*h*^2^) of olfactory behavior from the regressions of the cumulative response to selection onto the cumulative selection differential (Falconer and Mackay 1996). For the BUT selection regime (Supplemental material, Figure S2a), estimates of realized heritability for the three replicate BUT^+^ lines were: 1: *h*^2^*=*0.2301 ± 0.0130 (*P* < 0.0001), 2: *h*^2^*=*0.2560 ± 0.0066 (*P* < 0.0001), and 3: *h*^2^*=*0.1896 ± 0.0124 (*P* < 0.0001). Estimates of realized heritability for the three replicate BUT^-^ lines were: 1: *h*^2^*=*0.1748 ± 0.0297 (*P* < 0.0001), 2: *h*^2^*=*0.1890 ± 0.2620 (*P* < 0.0001), and 3: *h*^2^*=*0.1421 ± 0.0387 (*P* < 0.0016). For the CYC selection regime (Supplemental material, Figure S2b), estimates of realized heritability for the three replicate CYC^+^ lines were: 1: *h*^2^*=*0.4659 ± 0.0272 (*P* < 0.0001), 2: *h*^2^*=*0.3109 ± 0.0271 (*P* < 0.0001), and 3: *h*^2^*=*0.4614 ± 0.0284 (*P* < 0.0001). Estimates of realized heritability for the three replicate CYC^-^ lines were: 1: *h*^2^*=*0.1123 ± 0.0204 (*P* < 0.0001), 2: *h*^2^*=*0.1168 ± 0.0202 (*P* < 0.0001), and 3: *h*^2^*=*0.1044 ± 0.0206 (*P* < 0.0001). For both BUT and CYC selection regimes, the selection response was asymmetrical, with selection for PI^+^ stronger than selection for PI^-^.

Finally, we examined whether differences among the selected treatments indicated an experimentally induced shift in sensitivity, or if it indicated a binary ‘switch’ in the hedonic valence of odorants. If the former is true, we hypothesized a shift in the dose-response curves for the two selection treatments away from one another, with PI^+^ shifting toward higher concentrations and PI^-^ shifting toward lower concentrations; if the latter is true, there would be no concentration at which the odorant gave a negative (for PI^+^) or positive (for PI^-^) response. We found the former: a relative shift in the dose response curves of the selected treatments for both the BUT and CYC selection regimes, in the directions predicted (Figure 1G, H; Supplemental material, Table S5).

### Other organismal traits - responses correlated with selection

**Figure 2:**
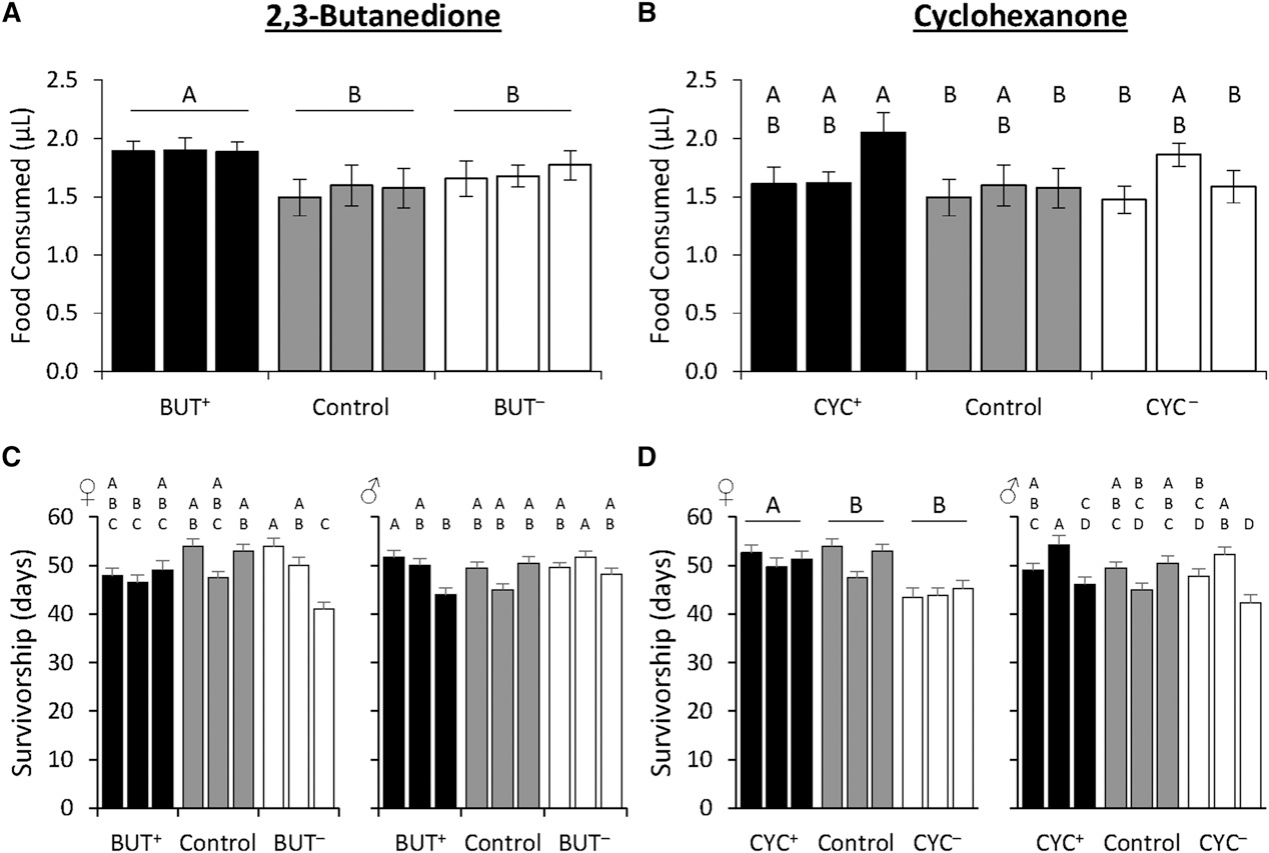
Correlated responses to selection for behavioral responses to 2,3-butanedione and cyclohexanone are sex-, odorant-, and trait-specific. (A and B) Mean food consumption for the three replicate PI^+^ (black bars), PI^-^ (white bars), and control treatments (gray bars) are shown. N = 12. (C and D) Mean survivorship for the three replicate PI^+^ (black bars), PI^-^ (white bars), and control (gray bars) are shown for each sex. N = 70. Data shown are means ± SE. Letters indicate *P* < 0.05 using Tukey’s *post hoc* test.

We tested whether other traits were correlated with selection by measuring food consumption, lifespan, and starvation resistance because olfaction has been shown to influence these traits (Libert *et al.* 2007; Gendron *et al.* 2014; Pool and Scott 2014; Wright 2016). Regarding food consumption, there were significant differences for the BUT selection regime (Figure 2A; Supplemental material, Table S4). The BUT^+^ treatment consumed significantly more than the BUT^-^ and control treatments, with an average of 1.89 ± 0.05 µL and 1.70 ± 0.05 µL per 24 hr, respectively, and 1.56 ± 0.05 µL for the control treatment. For the CYC selection regime, there was no difference among selection treatments (Figure 2B; Supplemental material, Table S4). With regard to lifespan, no significant differences were observed in the BUT selection regime among selection treatments (Figure 2C; Supplemental material, Table S6). But, regard the CYC selection regime, we found that the female CYC^+^ treatment lived for a significantly longer time than the female CYC^-^ and control treatments, with mean survival of 51.23 ± 0.92, 44.10 ± 0.98, and 49.67 ± 0.97 days, respectively (Figure 2D). Finally, for measurements of starvation resistance, although we found significant differences in survivorship among selection treatments for both selection regimes, we also observed a significant effect of line (Supplemental material, Figure S2; Supplemental material, Table S6).

### Olfaction – specificity of response to odorants

To determine whether selecting for valence behavior can cause it to be generalized from the selection odorant to a novel one, we measured behavioral responses of the BUT selected treatments to CYC, and vice versa. We hypothesized that lines selected for divergent responses to BUT, for example, would also exhibit divergent responses to CYC and vice versa. This hypothesis was based on the previous work examining the ligand specificity of the *D. melanogaster* odorant receptors, in which it was shown that structurally similar odorants typically have similar binding affinities and therefore will elicit similar behavioral responses (Khan *et al.* 2007). We observed a slight, but significant difference in olfactory behavior between BUT^+^ and BUT^-^ treatments in response to CYC. However, the same was not true for the opposite comparison (Supplemental material, Figure S4; Supplemental material, Table S7), suggesting that this hypothesis is only partially supported and generalization may be specific to the BUT selection regime.

For each selection regime, the potential for generalization was further examined by measuring the responses of the selection treatments and controls to a set of 12 additional ‘test’ odorants. These odorants were selected based on their structural similarity/dissimilarity with BUT and/or CYC. We tested whether (a) within a given selection regime there were test odorants that produced responses in the PI^+^ and PI^-^ treatments that mirrored the responses to the selection odorants, which would indicate that the effects of selection included attributing hedonic valence to other, presumably similar, odorants, and whether (b) patterns of PI differ between BUT and CYC selection treatments, which could indicate that different genetic/neural circuitry was recruited during the response to these selection odors.

**Figure 3:**
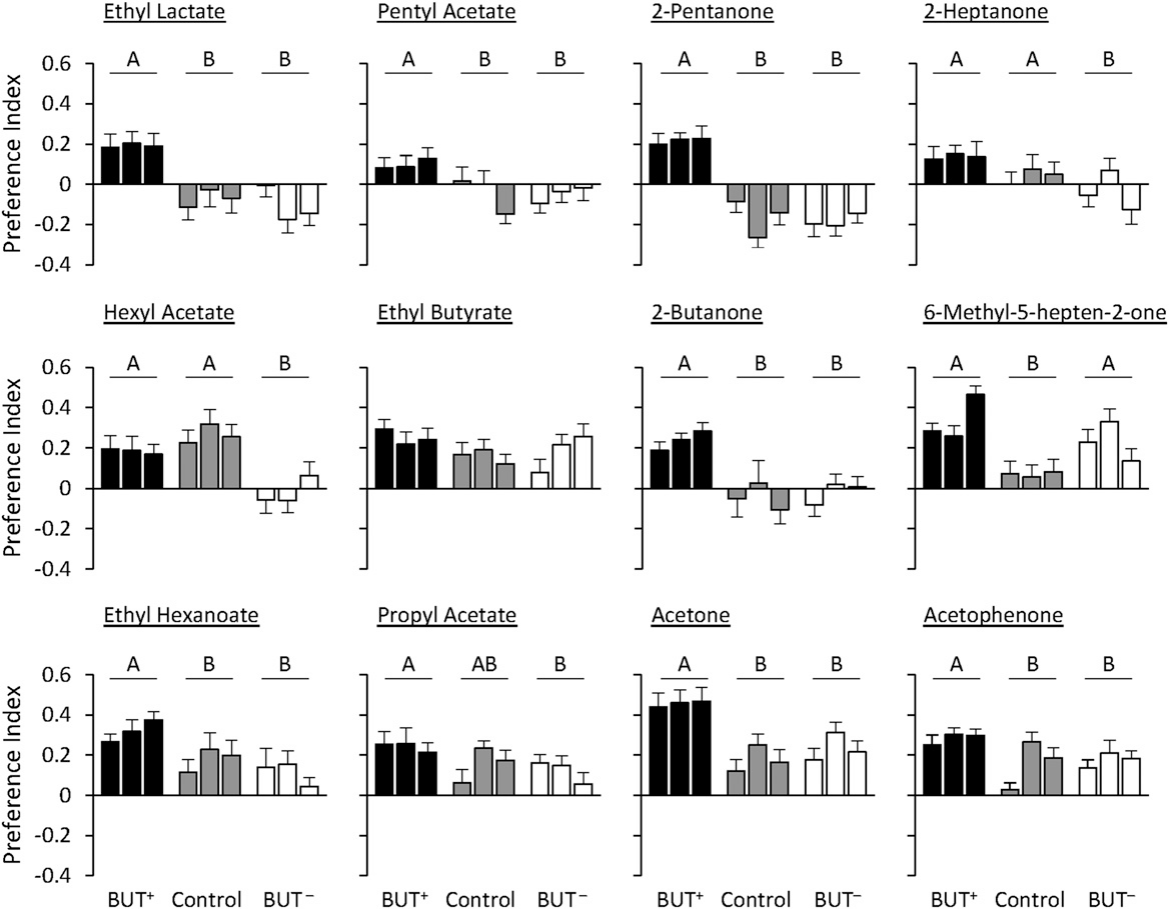
Olfactory behavioral responses of lines selected for 2,3-butanedione (BUT) to additional odorants. Behavioral responses for the three replicate BUT^+^ (black bars), BUT^-^ (white bars), and control treatments (gray bars) are shown for each sex. N = 20. Data shown are means ± SE. Letters indicate *P* < 0.05 using Tukey’s *post hoc* test.

**Figure 4:**
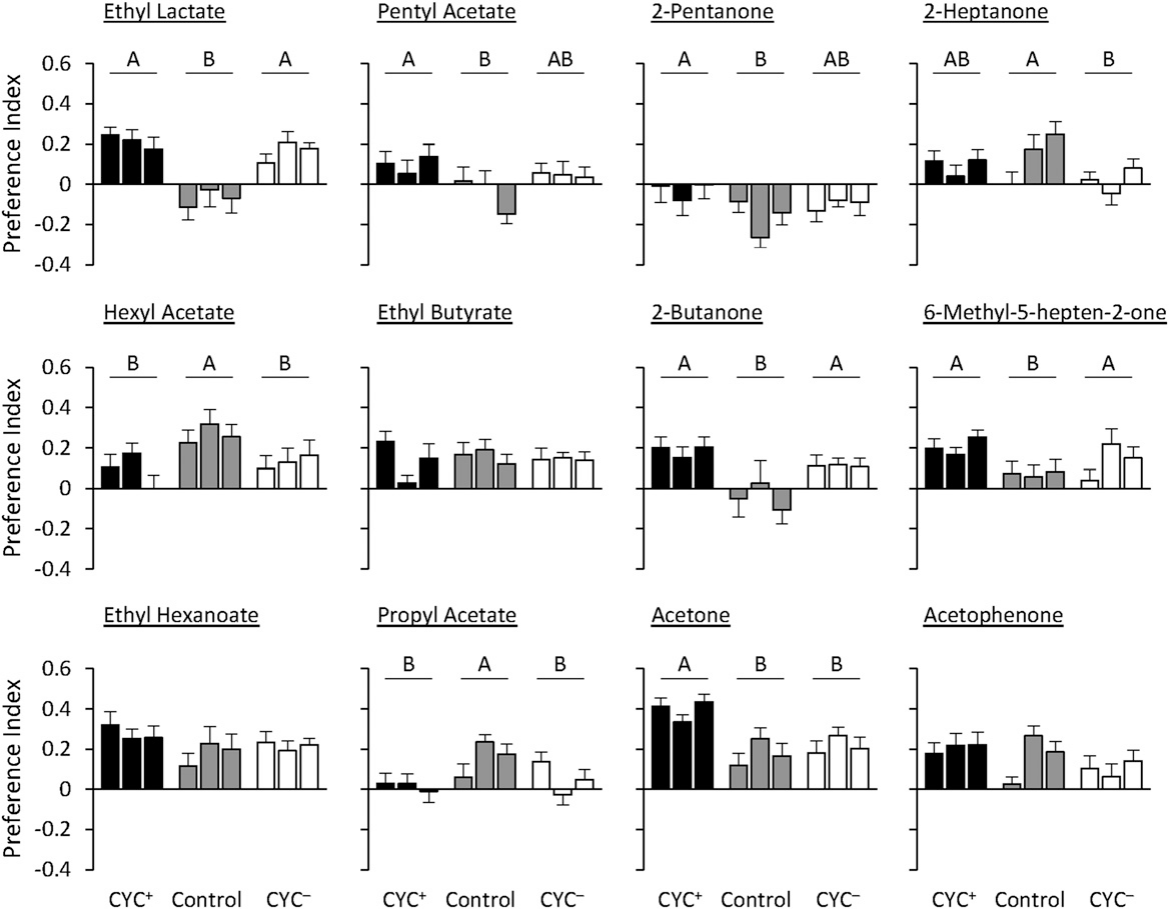
Olfactory behavioral responses of lines selected for cyclohexanone (CYC) to additional odorants. Behavioral responses for the three replicate CYC^+^ (black bars), CYC^-^ (white bars), and control treatments (gray bars) are shown for each sex. N = 20. Data shown are means ± SE. Letters indicate *P* < 0.05 using Tukey’s *post hoc* test.

Within the BUT selection regime, in five cases there was some evidence of generalization to a test odorant (ethyl lactate, pentyl acetate, 2-pentanone, 2-heptanone, hexyl acetate). Namely, the pattern of responses to these odorants was similar to their responses to BUT, in that the BUT^+^ and BUT^-^ treatments showed positive and negative responses, respectively, to these five test odorants (Figure 3; Supplemental material, Table S7). However, the responses of the BUT^+^ and BUT^-^ treatments to these odorants did not always differ from the control treatment (usually the BUT^-^ line). Nevertheless, responses to these five odorants are markedly different from responses to the other seven test odorants, which were nearly uniformly attractive in both selection regimes, and thus showed no evidence of a generalization effect. In the case of the CYC selection regime, there was no test odorant for which the PI of both CYC^+^ and CYC^-^ treatments were different in both mean and sign (Figure 4; Supplemental material, Table S7). In fact, in all but one case, the test odorants elicited attraction in both the CYC^+^ and CYC^-^ treatments (exception: 2-pentanone was aversive to both selection lines and control).

The PI patterns of BUT, CYC, and control treatments for two odorants, ethyl lactate and 2-pentanone, differ by selection regime. Responses of the PI^-^ treatments to ethyl lactate were opposite in valence (BUT^-^ were repelled, CYC^-^ were attracted), as were responses of the PI^+^ treatments to 2-pentanone (BUT+ were attracted, CYC^+^ were repelled). Overall, these comparisons show that the mechanisms underlying responses to novel test odorants are, in some cases, differentially affected by different selection odors.

**Figure 5:**
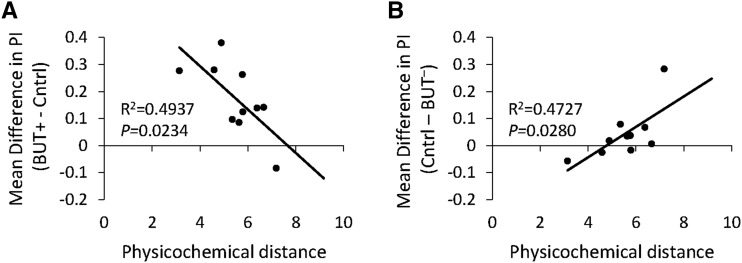
Linear regression analyses reveal a significant correlation between mean PI and physicochemical similarity among lines selected for behavioral responses to 2,3-butanedione (BUT). (A) There is a significant negative correlation between PI and physicochemical distance. The mean difference in PI was calculated by subtracting the mean PI of the three replicate control lines from the mean PI of the three replicate BUT^+^ lines. (B) There is a significant positive correlation between PI and physicochemical distance. The mean difference in PI was calculated by subtracting the mean PI of the three replicate BUT^-^ lines from the mean PI of the three replicate control lines. Physical distance was calculated as described in Haddad *et al.* (2008).

To determine whether generalization of valence from the odorant used for the selection regime(s) to a novel one can be explained by the physical similarity between the two, we calculated the physicochemical distance. For the BUT selection regime there was a significant negative correlation between ΔPI^+^ and physicochemical distance (Figure 5A). This indicates that the more similar the test and selection odorants are, the more likely the flies are to respond similarly to them (*P* = 0.0234). There was a significant positive correlation between ΔPI^-^ and physicochemical distance (Figure 5B), again showing that if the two odorants are similar, the flies are likely to respond similarly (*P* = 0.0280). In contrast, for the CYC selection treatment there were no significant correlations between ΔPI^+^ or ΔPI^-^ and physicochemical distance (Supplemental material, Figure S5), suggesting that the behavioral responses to the test odorants are not associated with their physical similarities to CYC.

### Transcriptional response to selection for odor-guided behavior

To examine the genetic mechanisms that contribute to attractive and aversive behavioral responses, we conducted RNA-seq analyses on both BUT and CYC selected lines and unselected controls. Differentially expressed genes were identified from whole heads, so as to include in the analysis genes expressed in both the brain and the peripheral olfactory organs. We obtained a total of 1,085,658,389 100-bp reads from 30 cDNA libraries (Supplemental material, Table S8). After quality filtering, 95.74% could be aligned to the *Drosophila melanogaster* genome and 96.88% of these reads mapped uniquely. Of the 17,471 annotated genes (Attrill *et al.* 2016), 8,765 genes had at least one read per million in at least half the samples (Supplemental material, Table S9). This set of genes was used for subsequent analyses.

**Figure 6:**
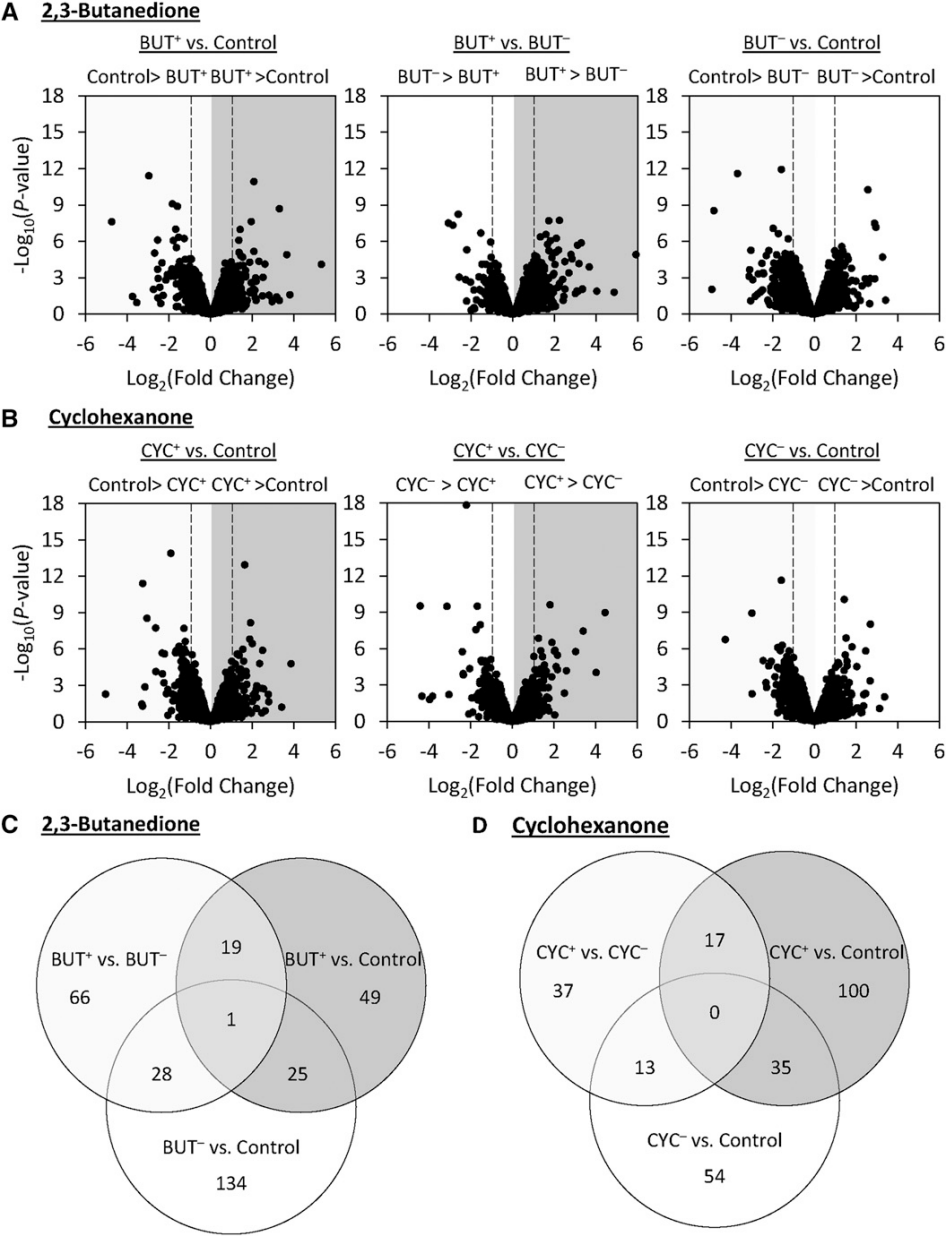
RNA-seq analyses of lines artificially selected for behavioral responses to 2,3-butanedione or cyclohexanone, as well as unselected controls. (A and B) Volcano plots of RNA-seq results for each pairwise comparison. For each comparison, the selection treatment to the left of the > symbol is upregulated, whereas the selection treatment to the right is downregulated. Vertical dashed lines represent a twofold threshold. (C and D) Venn diagrams illustrating the number of differentially expressed genes that are either unique or overlap between each pairwise comparison.

For both selection regimes, we made three comparisons of gene expression (1) PI^+^ *vs.* control, (2) PI^+^ *vs.* PI^**-**^, and (3) PI^-^ *vs.* control. In the BUT selection regime, 94, 114, and 188 genes were significantly differentially expressed in the BUT^+^ *vs.* control, BUT^+^ *vs.* BUT^-^, and BUT^-^ *vs.* control comparisons, respectively (Figure 6A; Supplemental material, Table S10-S12). In the CYC selection regime, a total of 152, 67, and 102 genes were significantly differentially expressed in the same respective comparisons (Figure 6B; Supplemental material, Table S13-S15). We found that 20 and 17 genes were shared between the PI^+^ *vs.* control and PI^+^ *vs.* PI^-^ comparisons in the BUT and CYC selection regimes respectively (Figure 6C,D), which may be associated with positive valence. In PI^+^ *vs.* PI^-^ and PI^-^ *vs.* control comparisons there were 29 and 13 genes shared in the respective BUT and CYC selection regimes (Figure 6C,D), comprising genes that may contribute to negative valence. Finally, in the BUT selection regime, only one gene was differentially expressed in all comparisons regardless of selection for PI^+^ or PI^-^ behavioral responses (Figure 6C), suggesting that this gene may contribute to generalized changes in olfactory behavior regardless of hedonic value. No such genes were identified in the CYC selection regime.

**Figure 7:**
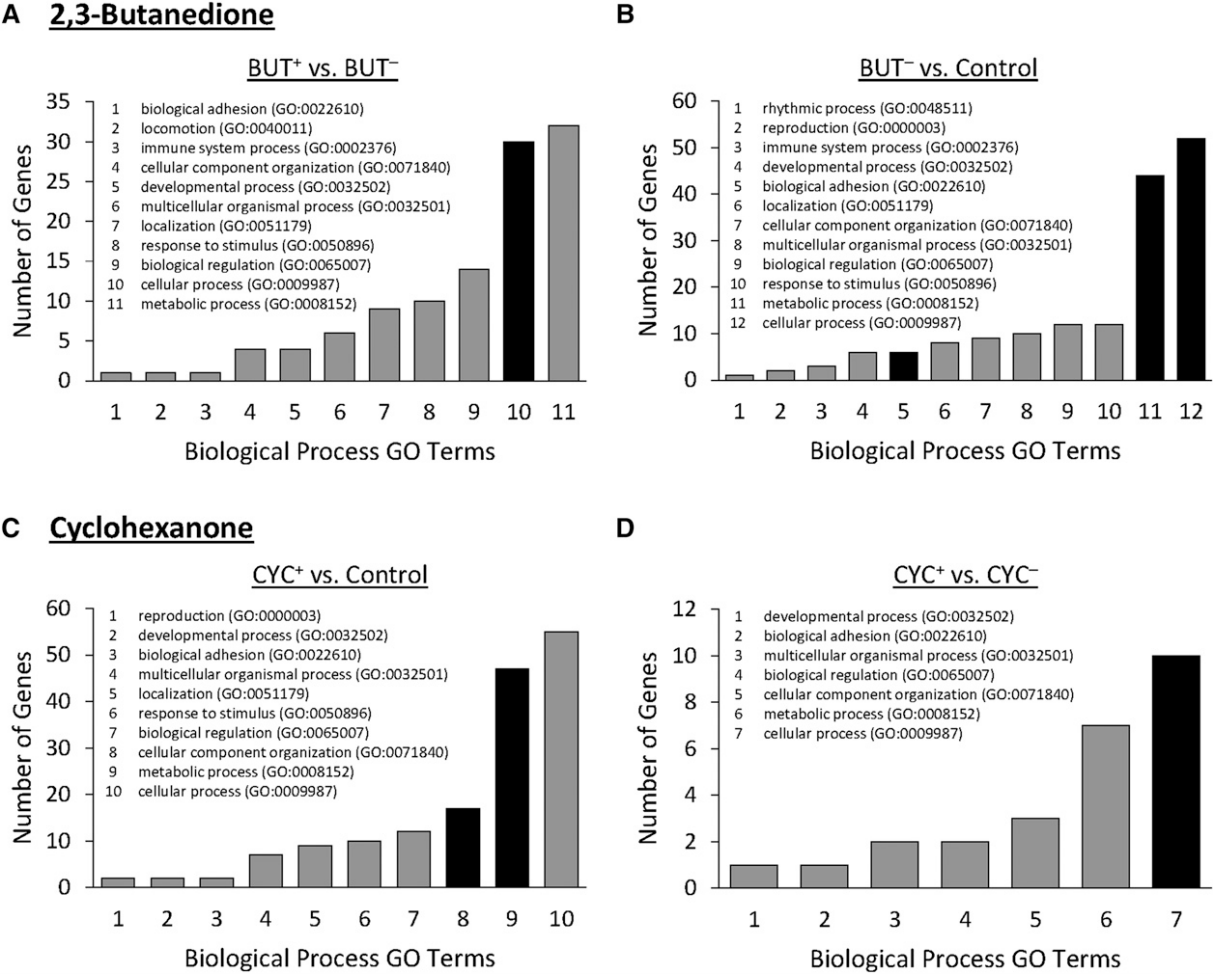
Biological Process Gene Ontology (GO) enrichment analyses identify GO terms associated with selection for behavioral responses to 2,3-butanedione (A and B) and cyclohexanone (C and D). Of the 6 pairwise comparisons shown in Figure 6, the ones with significant enrichment are shown here, which include: (A) BUT^+^ *vs.* BUT^-^, (B) BUT^-^ *vs.* Control, (C) CYC^+^ *vs.* Control, and (D) CYC^+^ *vs.* CYC^-^. The numbers correspond to the listed GO terms nested within each graph. For each comparison, the black bars indicate GO terms that are significantly enriched.

For each selection regime, the differentially expressed genes were annotated for specific Gene Ontology (GO) terms. We then assessed whether there was an overrepresentation of specific GO term(s) in the differentially expressed gene sets (Figure 7; Supplemental material, Table S16). For the BUT^+^ *vs.* BUT^-^ and BUT^-^ *vs.* control comparisons, we found significant overrepresentation for the biological process GO term, ‘sulfur compound metabolic process’ which is a subclass of the ‘cellular process’ GO term (Figure 7A,B). Included within this term are genes belonging to the glutathione S-transferase (Gst) family (BUT^+^ *vs.* BUT^-^: *GstD1*, *GstD3*, *GstD10*, *GstE5*, *GstE8*; BUT^-^ *vs.* control: Cystathionine β-synthase, *GstD9*, *GstD10*, *GstE5*, *GstE8*, *GstE14*, *GstZ2*). The terms ‘cell-cell adhesion’ and ‘translation’ were also overrepresented in the BUT^-^ *vs.* control comparison (Figure 7B), which are subclasses of the ‘biological adhesion’ and ‘metabolic process’ GO terms, respectively. For the CYC selection regime the terms ‘RNA metabolic process’ and ‘nitrogen compound metabolic process’- both of which are a subclass of the ‘metabolic process’ GO term, as well as ‘cellular component biogenesis’- a subclass of ‘cellular component organization’ GO terms, were significantly overrepresented in CYC^+^ compared to control (Figure 7C). Moreover, we again found significant overrepresentation of the GO term ‘sulfur compound metabolic process’ in the CYC^+^ *vs.* CYC^-^ comparison, as well as the term ‘cellular amino acid metabolic process’, both belonging to the subclass ‘cellular process’ (Figure 7D). Included in these two terms are once more genes belonging to the glutathione S-transferase family, and include *GstD3*, *GstD8*, *GstD9*, and *GstD10*. In both selection regimes, the directionality of differential expression relative to PI depended on the *Gst* gene. For example, *GstD10* is down-regulated in the BUT^+^ selection treatment (relative to BUT^-^) and up-regulated in the BUT^-^ and CYC^-^ selection treatments (relative to the control and CYC^-^ lines respectively), while *GstE8* is up regulated in the BUT^+^ selection treatment (relative to BUT^+^) and down-regulated in the BUT^-^ selection treatment (relative to control). No significant overrepresentation was observed for the remaining BUT^+^ *vs.* control or CYC^-^ *vs.* control pairwise comparisons (Supplemental material, Figure S6). Additionally, no significant overrepresentation of the terms ‘molecular function’ or ‘cellular component’ was found for differentially expressed genes of either selection regime (Supplemental material, Figure S7, Figure S8).

## Discussion

### Summary

We observed a significant divergence in behavioral responses to both BUT and CYC, and the responses were bisymmetric, with shifts in the dose response curves between the selected groups for both the BUT and CYC selection regimes. Other traits, namely food consumption and lifespan, varied with selection regime and, at times, sex. Generalization of the effects of selection to other odorants differed between the two selection regimes, and was only detected in the BUT selection regime. An analysis of gene expression of both BUT and CYC selection regimes subsequently identified multiple differentially expressed genes. In particular, a functional enrichment of the GO term ‘cell-cell adhesion’ as well as ‘sulfur compound metabolic process’ was found, the latter including genes belonging to the glutathione S-transferase family. These genes have been previously implicated in the termination of signaling in the peripheral olfactory organs (Vogt 2005).

### Selection effects on lifespan

Like previous studies (Libert *et al.* 2007; Poon *et al.* 2010; Gendron *et al.* 2014; Pool *et al.* 2014; Waterson *et al.* 2014; Sachse and Beshel 2016; Wright 2016), we found links between olfaction and both lifespan and food consumption. Females under the CYC selection regime were affected in lifespan; the CYC^+^ females had longer lifespan than the control and CYC^-^ females. This is in line with the findings by Libert *et al.* (2007) that loss-of-function mutations in chemosensory genes, specifically in the co-receptor ORCO, reduce neurophysiological and behavioral responses to many odorants and prolong *Drosophila* lifespan, and by Ostojic *et al.* (2014) that different gustatory receptor mutants vary in their effects on *Drosophila* lifespan. On the other hand, Brown *et al.* (2017) used the same methodology as in the present study and found no changes in lifespan after selection for behavioral responses to esters and aromatics. We speculate that the difference between that result and the ones herein are due to the fact that multiple mechanisms influence lifespan and because sensory input is subject to modification by these mechanisms (Russell and Kahn 2007; Ostojic *et al.* 2014; Waterson *et al.* 2014). Also, environmental factors, such as food composition, can affect lifespan (Skorupa *et al.* 2008; Grandison *et al.* 2009).

### Selection effects on food consumption

Chemosensation is also directly tied to food consumption (Pool *et al.* 2014; Sachse and Beshel 2016; Wright 2016). For instance, in Brown *et al.* (2017), selection for responses to two aromatic compounds led to changes in food consumption. The tested aromatics elicit responses from OSNs on the maxillary palp and given the organ’s proximity to the labellum, it has been hypothesized to play a role in taste enhancement (Shiraiwa 2008). In the present study the BUT^+^ selection treatment consumed more than the BUT^-^ and control treatments. BUT elicits responses from a large number of different OSNs (de Bruyne *et al.* 2001; Hansson *et al.* 2010), including those located on the maxillary palp (de Bruyne *et al.* 1999). Furthermore, of the genes that were differentially expressed among BUT selection treatments, 21 of them were previously identified in a GWA analysis of food consumption (Garlapow *et al.* 2017).

### Mechanisms underlying selection effects on hedonic valence

The present study found variation in behavioral responses to an odorant at a single concentration, and subsequent selection lead to increased attraction or aversion. The mechanisms underlying these shifts are likely complex and depend on the chemical compound used for selection, and on the direction of selection. Specifically, the PI^-^ flies under both selection regimes became more sensitive (in the behavioral sense), effectively shifting their dose-response curves to the left (compared to controls; Figure 1G,H). Likewise, the PI^+^ flies displayed the opposite, with a slight shift to the right of controls. More specifically, we observed a shift in the concentration at which valence switches from positive to negative. Different mechanisms have been reported to underlie behavioral valence, including at least one that depends on sensory sensitivity. For instance, it is well known that attractive responses often switch to aversive at high odorant concentrations, as we have seen here. This aversion can be caused by the recruitment, at high concentrations, of lower-affinity olfactory receptor neurons that initiate the reversal; the activity of a glomerulus innervated by such sensory neurons was found to be necessary and sufficient to switch the behavioral valence of vinegar from attractive to aversive (Semmelhack and Wang 2009; see also Knaden *et al.* 2012). This mechanism of determining valence is thus dependent on the relative sensitivity of different peripheral receptors, but odorant valence may also be determined more centrally. Knaden *et al.* (2012) showed the valence of certain odorants to be more closely correlated with the output from glomeruli to higher brain areas, than it is with the input to glomeruli from the periphery. Glomerular output is heavily dependent on interglomerular interactions, mediated by local interneurons (Wilson 2013), and so valence specified at this level may be somewhat independent of peripheral receptor sensitivity. Both of these valence mechanisms are likely to be naturally variable and thus provide the background necessary for selective divergence, and indeed there appears to be an intrinsic, genetic component to attraction. Ruebenbauer *et al.* (2008) found that, particularly for single synthetic compounds as were used in the present study, there are differences in affinity for attractive odors between different strains of *D. melanogaster*. This was not due to differences in peripheral sensitivity, as determined by electroantennogram. It is notable, however, that although degree of attraction differed, strains did not differ in the sign of hedonic valence over the concentrations tested. Nevertheless, behavioral valence can depend on the concentration-dependent activation of specific ‘aversive’ glomeruli, on within-antennal lobe processing prior to ascension to higher brain centers, and/or decisions made within higher centers, and any of these may vary between strains. Thus, the potential flexibility therein may explain the complex differences we found in the effects of selection for the two odorants; namely, that the BUT selection treatment responds similarly to similar but novel test odorants, but the CYC selection treatment does not (Figures 3-5, S4).

### Genes differentially expressed after selection

The results of our RNA-seq experiment identified a number of genes differentially expressed between the PI^+^, PI^-^, and the unselected control treatment. Many of these genes were not previously identified as mediating olfactory behavior, suggesting a new role for these genes. A previous GWA study identified 110 genes associated with odor-guided behavior (Brown *et al.* 2013; Swarup *et al.* 2013; Arya *et al.* 2015), 31 of which were differentially expressed among lines selected for behavioral responses to 4-ethylguaiacol (Brown *et al.* 2017). These genes were identified despite odorant identity and valence, suggesting that they may function in generalized behavioral responses to odor. Of particular note is Odorant-binding protein 56h (*Obp56h*). This gene is expressed in select sensilla on the antenna and gustatory organs (Galindo and Smith 2001; Martin *et al.* 2013), and has been implicated in feeding and mating behaviors (Swarup *et al.* 2014; Shorter *et al.* 2016). Here, we found that expression of *Obp56h* was upregulated in both the BUT^-^ and CYC^-^ selection treatments relative to the control treatment, suggesting it could play a role in aversive responses to odor. Consistent with this conclusion is the observation that silencing expression of *Obp56h* using RNAi increases behavioral responses to acetophenone, another ketone (Swarup *et al.* 2011). The RNA-seq results also speak to the extent to which any behavioral difference between selection groups are likely to have arisen from a difference in peripheral sensory sensitivity. This is because such a difference may be caused by a difference in expression levels of odorant receptors within the olfactory sensory neurons, or by a difference in the number of specific olfactory sensory neurons (which is correlated with expression levels: Li *et al.* 2013; Crowley-Gall *et al.* 2016), or both. The fact that we did not find significant differences in odorant receptor gene expression levels in RNA-seq analyses of either selection regime limits support for either of these options.

### Gene ontology of differentially expressed genes

We found overrepresentation of several gene ontology categories, including *cell-cell adhesion*, *sulfur compound metabolic process*, and *cellular amino acid metabolic process*. Cell-cell adhesion molecules have been previously implicated in OSN axon guidance during development, guiding them to appropriate dendrites of projection neurons (Hummel and Zipursky 2004). Indeed, one of the genes listed under this GO term, Hig-anchoring scaffold protein (*Hasp*) is an extracellular matrix protein located in the axon terminals of olfactory projection neurons and is involved in synaptogenesis (Nakayama *et al.* 2016). Additionally, the *cell-cell adhesion* GO term was significantly enriched in a GWA study of behavioral responses to a panel of 14 odorants (Arya *et al.* 2015). Two genes that were identified in Arya *et al.* (2015), starry night (*stan*) and wing blister (*wb*) were associated with variation in behavioral responses to two ketones (2-heptanone and D-carvone, respectively). These genes were also identified in this study examining responses to BUT and CYC, compounds belonging to the same chemical class.

### Possible role of GSTs in valence

Genes comprising the *sulfur compound metabolic process* GO category include Glutathione S-Transferases (GSTs), enzymes that conjugate glutathione to electrophilic compounds, rendering the resulting compound more water soluble and easier to eliminate (Enayati *et al.* 2005). Specifically, the GST gene family consists of several classes, with the GST Delta (GSTD) and Epsilon (GSTE) classes being present exclusively in insects (Chelvanayagam *et al.* 2001; Low *et al.* 2007). These enzymes are hypothesized to eliminate toxic compounds, conferring resistance to synthetic insecticides and natural plant allelochemicals (Li *et al.* 2007; Després *et al.* 2007). For example, *GSTD1* mediates insecticide metabolism and in *D. melanogaster* increased *GSTD1* is associated with DDT resistance (Tang and Tu 1994; Low *et al.* 2010). *GSTD1* has also been implicated in the evolution of host plant adaptation in species of cactophilic *Drosophila* (Matzkin *et al.* 2006; Matzkin 2008; López-Olmos *et al.* 2017). In insect olfaction, GSTs are hypothesized to modulate insect perception by functioning in odorant degradation underlying the termination of odorant signaling in the antenna, and enabling the insect to quickly respond to rapid changes in environmental volatiles (Vogt *et al.* 1985; Rogers *et al.* 1999). In the present study, genes in the GST family were differentially expressed between the selection groups in both the BUT and CYC selection regimes. With the exception of *GstE14*, the GST genes differentially expressed in this study were identified in the antennal transcriptomes of *D. melanogaster* males and females, regardless of female mating status (Riveron *et al.* 2013; Younus *et al.* 2014). Moreover, the list of GSTs differentially expressed is partially overlapping for the BUT and CYC selection regimes and in both regimes, the direction of differential expression relative to PI depended on the gene. The importance of this overlap and direction as it relates to olfactory behavior and cross-behavioral patterns remains to be determined because few GSTs have been extensively characterized. But interestingly, select chemical compounds can inhibit and others induce different GSTs (Yu 1996; Yu and Abo-Elghar 2000; Willoughby *et al.* 2006; Gonzalez *et al.* 2018). Future work is needed to test the association between GST expression, insect perception, and behavior to understand their role in the evolution of olfactory behavior. Nevertheless, our findings highlight a potential role for this class of genes in the evolution of hedonic valence to ecologically relevant volatile compounds.
